# APOE protects against severe infection with *Mycobacterium tuberculosis* by restraining production of neutrophil extracellular traps

**DOI:** 10.1371/journal.ppat.1013267

**Published:** 2025-06-16

**Authors:** Dong Liu, Dat Mai, Ana N. Jahn, Tara A. Murray, John D. Aitchison, Benjamin H. Gern, Kevin B. Urdahl, Alan Aderem, Alan H. Diercks, Elizabeth S. Gold

**Affiliations:** 1 Center for Global Infectious Disease Research, Seattle Children’s Research Institute, Seattle, Washington, United States of America; 2 Department of Pediatrics, University of Washington, Seattle, Washington, United States of America; 3 Department of Immunology, University of Washington, Seattle, Washington, United States of America; 4 Virginia Mason Franciscan Health, Seattle, Washington, United States of America; Columbia University, UNITED STATES OF AMERICA

## Abstract

Mice lacking apolipoprotein E (APOE, *Apoe*^*-/-*^ mice) on a high cholesterol (HC) diet are highly susceptible to infection with *Mycobacterium tuberculosis* (Mtb) but the underlying immune dysregulation has been unclear. While neutrophils are often the predominant cell type in the lungs of humans with severe tuberculosis (TB), they are relatively scarce in the lungs of some strains of mice that are used to study the disease. The neutrophil levels in the lungs of Mtb-infected *Apoe*^*-/-*^ HC mice are very high, and thus studies in this model offer the opportunity to examine the role of specific neutrophil functions in the pathology of severe TB. We determined that depleting neutrophils, depleting plasmacytoid dendritic cells (pDCs), or blocking type I interferon signaling improved the outcome of TB in *Apoe*^*-/-*^ HC mice. We also demonstrated that blocking the activation of peptidylarginine deiminase 4 (PAD4), an enzyme critical to NET formation, leads to fewer NETs in the lungs and dramatically improves the outcome of TB in *Apoe*^*-/-*^ HC mice without affecting the number of neutrophils in the lung. We found that the transcriptional profile of neutrophils in Mtb-infected *Apoe*^-/-^ HC mice is biased towards a state that resembles the “N2” phenotype that has been defined in cancer models and has been implicated in matrix degradation and tissue destruction. Our observations strongly suggest that the state of the neutrophil when it encounters the Mtb-infected lung is one of the main drivers of severe disease and implies that targeted interventions that alter specific states or functions, such as the production of NETs, may improve outcome while preserving sufficient capacity for host-defense.

## Author summary

Tuberculosis (TB) is a global health emergency of massive proportions with an annual burden of approximately 10.8 million new cases of active disease and 1.2 million deaths in 2023. Although there is an increasing appreciation for the role of neutrophils in the pathology of severe TB, the specific neutrophil functions that are detrimental rather than beneficial to control of the disease have not been fully defined. Neutrophils are innate immune effector cells that engage multiple mechanisms to counter invading microbes, including the formation of neutrophil extracellular traps (NETs), and our study indicates that this process is a significant contributor to poor control of Mtb infection during severe disease, and that specifically blocking NET formation can improve outcomes. Thus, our study suggests NET formation as a target for host-directed therapies to treat severe TB.

## Introduction

Apolipoprotein E (APOE) is a member of a group of lipid binding proteins that plays an important role in lipid transport and metabolism through its interaction with multiple lipoprotein particles, and acts as a ligand for their receptor-mediated clearance [1]. One of the main receptors for APOE is the low-density lipoprotein receptor (LDLR) and binding of APOE to the LDLR leads to the clearance of lipoprotein particles from the circulation. While several differences in the models exist, both strains of mice develop similar levels of hypercholesterolemia on high cholesterol diets and, under these conditions, both develop atherosclerotic plaques [2–4]. In addition to its role in lipid transport, APOE has been implicated in inflammatory responses [5] and has also been shown to play a role in several infectious diseases [6–11]. An early study found that *Apoe*^*-/-*^ mice on a high-cholesterol (HC) diet are highly susceptible to Mtb-infection and that the susceptibility is increased with increasing hypercholesterolemia [12]. Surprisingly, *Ldlr*^*-/-*^ mice with similar levels of hypercholesterolemia to *Apoe*^*-/-*^ mice were relatively resistant to Mtb, mounting a timely immune response and demonstrating a similar capacity for controlling the bacteria as wild-type (WT) C57BL/6 (B6) mice [13]. Because *Apoe*^*-/-*^ mice develop necrotic lesions containing large numbers of neutrophils, similar to those seen in humans with severe tuberculosis [14], we sought to use this model system to uncover factors leading to severe tuberculosis using *Ldlr*^*-/-*^ mice as a control for any confounding effects of the hypercholesterolemia.

While neutrophils are often the most abundant cell type in the lungs of patients with severe tuberculosis, comprising 38–86% of cells recovered from cavitary lesions, sputum, or BAL [14], they are a relatively small fraction (~5%) of the responding immune cell population in the most commonly used (and relatively resistant) mouse model of tuberculosis, B6 mice infected with Mtb H37Rv [15]. In more susceptible strains, excessive neutrophil recruitment has been shown to be detrimental to the control of Mtb, and depleting neutrophils or blocking their recruitment to the lung partially reverses the phenotype [16–18]. Several factors have been shown to play a role in recruiting neutrophils to the lungs of mice infected with Mtb. Type I interferon is generally considered to be detrimental to control of Mtb infection and several studies have proposed a direct link between excessive type I interferon and neutrophil activation [19–23]. Both pDCs and macrophages have been shown to produce type I interferon in the lungs of Mtb-infected mice and non-human primates [18]. While pDCs represent a relatively small proportion of immune cells in the lung, it has been demonstrated that they are major producers of type I interferon [24–26] that can be activated via TLR7 and TLR9 recognition of extracellular DNA [26,27].

Neutrophils are short-lived innate immune effectors that engage multiple mechanisms to counter invading microbes, including the formation of “neutrophil extracellular traps” (NETs). Formation of NETs is an active process involving citrullination of histones, chromatin decondensation, and extravasation of DNA and associated proteins [28]. Classically, NET formation depends on activation of the enzyme peptidylarginine deiminase 4 (PAD4) which traffics from the cytoplasm to the nucleus to promote citrullination of histones, and inhibiting PAD4 has been shown to prevent NET formation [28]. Although PAD4-independent NET formation has been described [29], NET formation in Mtb-infected neutrophils is at least partially dependent on PAD4 [22,30]. It has been increasingly appreciated that neutrophils are not a homogenous cell type but can be polarized towards functionally different states. In the context of cancer, it has been proposed that tumor associated neutrophils (TANs) can be polarized into N1 (immunostimulatory) or N2 (immunosuppressive) phenotypes which are considered to be anti- or pro-tumorigenic respectively. N1 neutrophils produce high levels of inflammatory cytokines and other molecules that activate T cells while N2 neutrophils produce multiple matrix-metalloproteases (MMPs) which promote tissue remodeling and angiogenesis [31–33]. Neutrophil polarization has been much less well studied in the context of infectious disease generally and in the context of TB specifically. However, a recent report suggests that in human TB patients, higher expression of N1 compared to N2 surface markers correlates with increased lymphocytic responses to Mtb antigens [34].

We observed excessive neutrophil recruitment to the lungs of Mtb-infected *Apoe*^*-/-*^ HC mice as compared to Mtb-infected wild-type (B6) or *Ldlr*^*-/-*^ HC mice and found that depleting neutrophils in *Apoe*^*-/-*^ HC mice partially reversed the excess bacterial burden in these mice. We also demonstrated that depleting pDCs or blocking type I interferon signaling partially improved the outcome of Mtb infection. Most strikingly, we showed that inhibiting PAD4, and thus decreasing NET formation, returned the bacterial burden in the lungs of *Apoe*^*-/-*^ HC mice back to that seen in B6 mice and significantly increased their survival without decreasing the number of neutrophils in the lung. We found that there was an enrichment of N2-like neutrophils in the lungs of *Apoe*^*-/-*^ HC mice, compared to control *Ldlr*^*-/-*^ and B6 HC mice. Thus, our study suggests that the state of the neutrophil when it encounters the Mtb-infected lung is one of the main drivers of severe disease and implies that targeted interventions that alter specific states or functions, such as the production of NETs, may improve outcome while preserving sufficient capacity for host-defense.

## Results

### *Apoe*^*-/-*^ HC mice are highly susceptible to infection with Mtb

**Fig 1:**
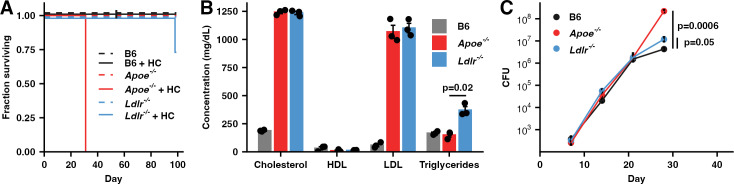
*Apoe*^*-/-*^ HC mice are highly susceptible to infection with Mtb. (**A**) Male mice of the indicated genotypes were fed either normal food or high-cholesterol food for two weeks and then infected with ~50 CFU Mtb H37Rv and maintained on their pre-infection diet. (n = 3-5 mice/group) (**B**) Serum cholesterol profiles at day 28 following infection of the indicated genotypes of mice fed HC food and infected with Mtb H37Rv as in (A). HDL = high-density lipoproteins, LDL = low-density lipoproteins. (n = 3 mice/group) (**C**) Bacterial burden in the lung measured by CFU counting for mice of the indicated genotypes at the indicated time points fed HC food and infected with Mtb H37Rv as in (A). (n = 5-7 mice/group) Bars/lines indicate mean; error bars indicate SEM. Significance analysis was performed using the two-sided Student’s t-test allowing for unequal variances (C).

To directly compare the susceptibility of hypercholesterolemic *Apoe*^*-/-*^ and *Ldlr*^*-/-*^ mice, we placed both on a HC diet for two weeks and then infected them via aerosol with approximately 50 CFU of Mtb H37Rv. Both male and female *Apoe*^*-/-*^ HC mice were significantly more susceptible to Mtb than sex and age matched *Ldlr*^*-/-*^ HC mice or B6 HC mice (Figs 1A and S1A) despite broadly similar serum cholesterol profiles of the two knockout strains (Figs 1B and S1B). *Apoe*^*-/-*^ HC mice control Mtb growth with similar efficiency to *Ldlr*^*-/-*^ HC or B6 HC mice for the first 21 days following aerosol challenge but subsequently lose control of the infection and by day 28 have nearly 10-fold higher bacterial burdens (Fig 1C). *Apoe*^*-/-*^ mice on normal chow are moderately hypercholesterolemic [3] and, while not hypersusceptible to infection with Mtb (Fig 1A), have an approximately 5-fold higher bacterial burden at day 28 post-infection (PI) than WT mice (S1C Fig).

### T cell priming is intact in *Apoe*^*-/-*^ HC mice

**Fig 2:**
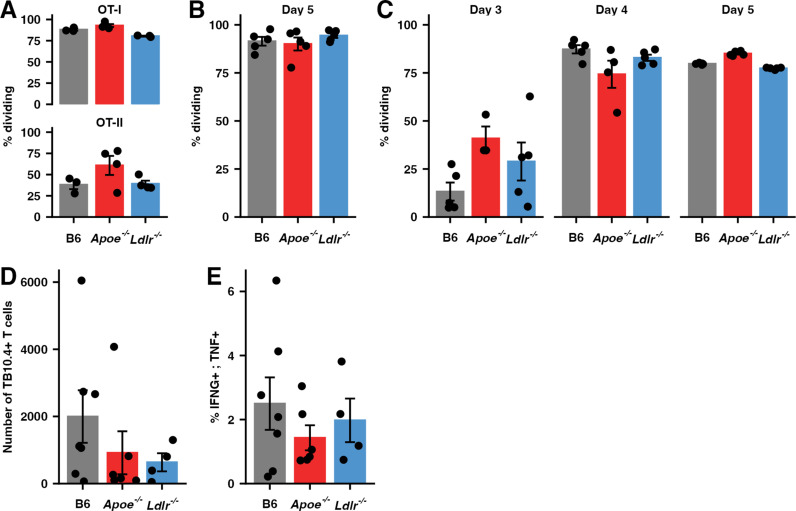
T cell priming is intact in *Apoe*^*-/-*^ HC mice. (**A**) Expansion of CFSE-labeled, CD8 (OT-I) or CD4 (OT-II) T cells specific for Ova peptides as measured by flow cytometry, shown as a percentage of cells dividing in the draining (mediastinal) lymph node, in mice of the indicated genotypes maintained on a HC diet at 4 days following intranasal inoculation with 2x10^8^ CFU BCG-Ova. (n = 3-4 mice/group) (**B**, **C**) Expansion of CFSE-labeled, ESAT-6 specific transgenic CD4 + T cells (C7) as measured by flow cytometry, shown as a percentage of cells dividing in the draining (cervical) lymph node, in mice of the indicated genotypes maintained on a normal (B) or HC (C) diet at the indicated days following inoculation with 10,000 CFU Mtb H37Rv in the dermis of the ear. (n = 3-5 mice/group) (**D**) Number of CD8 + TB10.4 + T cells in the lung parenchyma (defined by lack of labeling by an intravenous anti-CD45 antibody (IV-), see Methods) at day 19 following infection with ~50 CFU Mtb H37Rv in the indicated genotypes of mice maintained on a HC diet. (n = 7 mice/group) (**E**) Percentage CD8 + T cells in single-cell suspensions of lung tissue from mice in (D) producing both IFNG and TNF when restimulated with TB10.4 peptides assessed by intracellular staining and flow cytometry. (n = 7 mice/group) Bars indicate mean; error bars indicate SEM. Data are representative of 2-4 independent experiments. See S9 Fig for gating strategies.

In the first publication describing this model, the authors presented evidence, including impaired proliferation of CFSE labeled OT-II OVA-specific T cells stimulated in vivo with OVA-coated beads, suggesting that the extreme susceptibility of *Apoe*^*-/-*^ HC mice to Mtb infection results from defective T cell priming [12]. However, a recent study demonstrated that APOE deficiency in dendritic cells (DCs) enhances their ability to present antigens to CD4 T cells, resulting in more efficient T cell priming [35]. We performed a series of experiments to reconcile these disparate findings by evaluating DC and T cell function from *Apoe*^*-/-*^ mice. To test the ability of *Apoe*^*-/-*^ DCs to present model antigens to T cells in vivo, we adoptively co-transferred OVA-specific, CFSE-labeled OT-I and OT-II T cells into either *Apoe*^*-/-*^, *Ldlr*^*-/-*^ or B6 HC mice. 24hrs later, recipient mice were intranasally (IN) challenged with live recombinant BCG expressing OVA [36] (BCG-OVA). Four days later, mice were sacrificed and the expansion of OVA-specific T cells in the mediastinal lymph nodes was measured by CFSE dilution. There was no impairment in the ability of *Apoe*^*-/-*^ DCs to present OVA peptides to either the OT-I or OT-II T cells (Fig 2A). To examine the ability of *Apoe*^*-/-*^ DCs to present Mtb antigens in vivo we adoptively transferred CFSE labeled Mtb-specific (C7) CD4 T cells [37], which have been engineered to express T cell receptors specific for the Mtb antigen ESAT6, to *Apoe*^*-/-*^ mice fed either normal (Fig 2B) or HC food (Fig 2C). One day later, the mice were injected intradermally (ID) in the ear with 104 Mtb H37Rv [38] and T cell proliferation in the cervical lymph node was examined after inoculation. In both cases *Apoe*^*-/-*^*, Ldlr*^*-/-*^, and B6 DCs were equally effective at driving proliferation of exogenous T cells (Fig 2B and 2C).

We also assessed the number of functionally active, Mtb-specific T cells in the lungs of all three genotypes at day 19 PI with ~50 CFU of H37Rv, a time point where the adaptive immune system has begun to respond to Mtb but that precedes the divergence of bacterial burden (Fig 1C). We found no significant difference in the number of Mtb-specific CD8 (TB10.4 tetramer+) cells in the lungs of *Apoe*^*-/-*^ HC mice when compared to B6 and *Ldlr*^*-/-*^ HC mice (Fig 2D). Furthermore, there was no significant difference in the capacity of these cells to produce IFNG and TNF in response to ex vivo restimulation with Mtb-specific peptide (Fig 2E). While these experiments do not exclude a contribution of T cell function to the extreme susceptibility of *Apoe*^*-/-*^ HC mice they do suggest that innate priming of the adaptive response is broadly intact.

### Excessive NET formation contributes to the extreme susceptibility of *Apoe*^*-/-*^ HC mice

**Fig 3:**
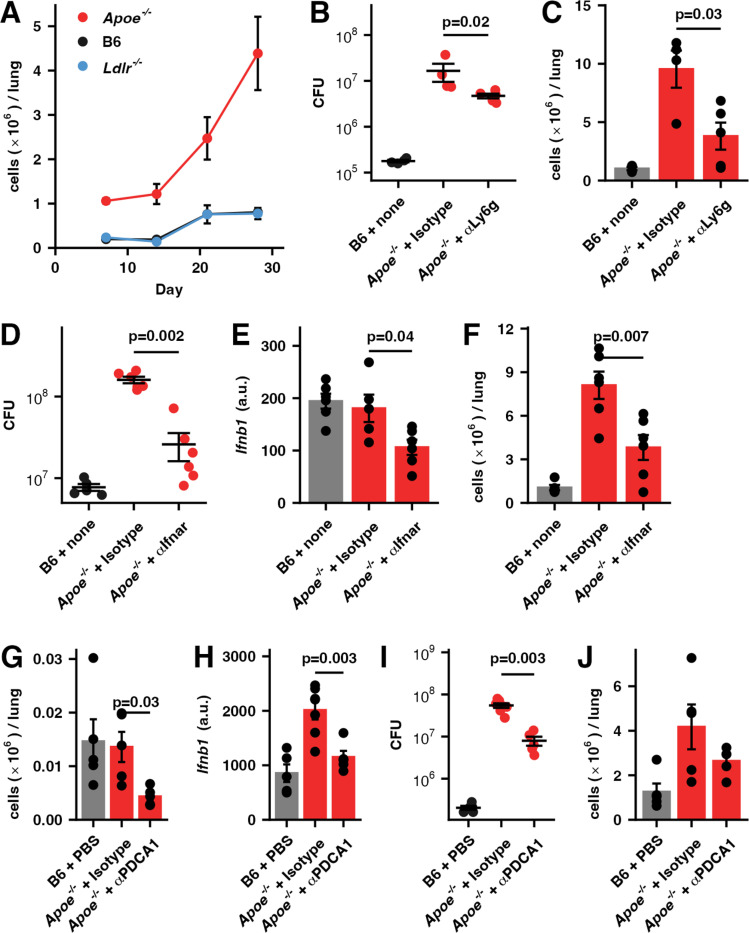
Neutrophils, pDCs, and type I interferon contribute to the susceptibility of *Apoe*^*-/-*^ HC mice. (**A**) The kinetics of neutrophil infiltration into the lungs of mice of the indicated genotypes maintained on a HC diet and infected with ~50 CFU H37Rv, as assessed by flow cytometry, expressed as the total number of neutrophils in the lung. (n = 5-7 mice/group) (**B-J**) *Apoe*^*-/-*^ or B6 mice were placed on a HC diet for two weeks, infected with ~50 CFU H37Rv via aerosol, and maintained on the diet for the entire experiment. *Neutrophil depletion:* (B) Pulmonary bacterial burden and (C) total neutrophil numbers in the lung at day 24 PI in the indicated treatments. *IFNAR blockade:* (D) Pulmonary bacterial burden, (E) expression of *Ifnb1* mRNA, and (F) neutrophil fractions of CD45 + cells in the lung at day 21 PI in the indicated treatments. *pDC depletion:* (G) Total numbers of pDCs in the lung, (H) expression of *Ifnb1* mRNA in the lung, (I) pulmonary bacterial burden at day 28 PI, and (J) total numbers of neutrophils in the lung. Bars/lines indicate mean; error bars indicate SEM. Data are representative of 2 independent experiments (n = 4-7 mice/group) (B-J). Significance analysis was performed using the two-sided Student’s t-test allowing for unequal variances (C, E-H, J) or the Wilcox rank-sum test (B,D,I). See S10 Fig for gating strategies.

We examined the pulmonary cellularity in *Apoe*^*-/-*^, *Ldlr*^*-/-*^, and B6 mice on a HC diet over the first 4 weeks of infection and observed that the most striking difference between genotypes was highly elevated levels of neutrophils in *Apoe*^*-/-*^ HC mice (Figs 3A and S2). As seen in other systems [16–18], antibody mediated depletion of neutrophils reduced bacterial burden in the lung (Fig 3B). *Apoe*^*-/-*^ HC have approximately five times as many neutrophils in the lung as controls prior to infection (S3A Fig), and while we were able to reduce these numbers, the overall level remained above that seen in the B6 animals (Fig 3C). There was no significant effect of the anti-Ly6g antibody on the absolute number of other immune cell types in the lung (S3B Fig).

In some models of severe TB (e.g., C3HeB/FeJ (“Kramnik”) mice [39], *Sp140-/-* mice [40], and mice depleted of GM-CSF [19]) the excess pathology is largely dependent on dysregulated type I interferon signaling, while in other models (i.e., *Nos2*^*-/-*^ and *Acod1*^*-/-*^ mice) the excess bacterial burden appears to be independent of type I interferon and has largely been attributed to dysregulated IL1 signaling [41]. To establish the role of type I interferon in this system we inhibited the type I interferon receptor (IFNAR) with a blocking antibody. This led to a significant decrease in bacterial burden (Fig 3D) and blocking IFNAR decreased the total amount of type I interferon and the total number of neutrophils in the lung (Fig 3E and 3F) with either no effect or a minimal effect on the absolute number of other immune cells in the lungs (S4 Fig).

pDCs are major producers of type I interferon [24,25], therefore, to test the hypothesis that they play a role in the susceptibility of *Apoe*^*-/-*^ mice to tuberculosis we examined the effect of depleting them using antibody treatment on the outcome of infection. As expected, depleting pDCs led to a decrease in *Ifnb1* expression (Fig 3G and 3H). Depleting pDCs significantly decreased bacterial burden in *Apoe*^*-/-*^ HC mice without significantly affecting the number of neutrophils in the lung (Fig 3I and 3J). The depletion antibody, anti-PDCA1, binds to bone marrow stromal cell antigen 2 (BST2), a receptor expressed on several cell types including DCs, mature B cells, and monocytes [42], however, we do not measure any significant decrease in these populations (S5 Fig). While we cannot formally rule out a contribution from these other cell types to the decrease in bacterial burden, these measurements, and the fact that expression of *Ifnb1* in the antibody-treated mice returned to WT levels (Fig 3H) suggest that the major effect of the treatment was to deplete pDCs.

**Fig 4:**
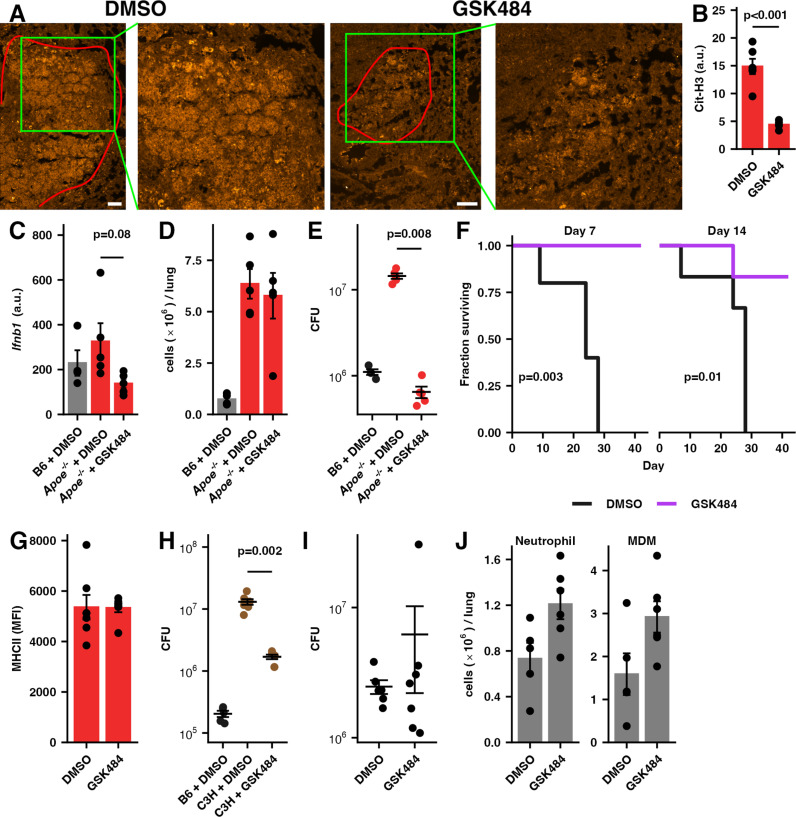
Restraining NET formation protects *Apoe*^*-/-*^ HC mice against severe tuberculosis. (**A**) Representative images of lung sections from Mtb H37Rv infected *Apoe*^*-/-*^ HC mice treated with GSK484 or vehicle daily from days 7-28 PI. Sections were labeled with anti-Cit-H3 antibody (orange) and imaged with confocal microscopy. Red outline indicates approximate lesion extent. Scale bar is 100 µm. (**B**) Quantification of the mean fluorescent signal of Cit-H3 labeling for 6 lesions from 3 mice from each condition in (A). (n = 6 lesions/group) (**C**) Expression of *Ifnb1* mRNA in the lung, (**D**) total number of neutrophils in the lung, (**E**) and bacterial burden at day 28 PI in mice treated as indicated. (n = 4-7 mice/group) (**F**) *Apoe*^*-/-*^ HC mice were infected as in (A) and treated with GSK484 or vehicle daily starting at either day 7 or day 14 PI. The fraction of mice surviving to day 40 is plotted. (n = 5-6 mice/group) (**G**) The expression of MHCII on MDM expressed as MFI assessed by flow cytometry from mice treated as in **(A)**. (n = 7 mice/group) (**H**) C3H mice were infected with ~50 CFU Mtb SA161 treated with GSK484 or vehicle daily starting at day 7 PI. Bacterial burden in the lung was measured by CFU at day 28 PI. (n = 6-7 mice/group) (**I**) B6 mice were infected with ~50 CFU Mtb H37Rv and treated with GSK484 or vehicle daily starting at day 7 PI. Bacterial burden in the lung was measured by CFU at day 28 PI. (n = 6-7 mice/group) (**J**) The number of neutrophils and monocyte-derived macrophages in the lung as measured by flow cytometry at day 28 PI in mice described in (**I**). Bars/lines indicate mean; error bars indicate SEM. Data are representative of two independent experiments (C-E, G). Significance analysis was performed using the two-sided Student’s t-test allowing for unequal variances (B,C,D,G,J), the Wilcox rank-sum test (E,H,I), or the Mantel-Haenszel test (F). See S10 Fig for gating strategies.

Neutrophils are short-lived innate immune effectors that engage multiple mechanisms to counter invading microbes, including the formation of NETs. Formation of NETs is an active process involving citrullination of histones, chromatin decondensation, and extravasation of DNA and associated proteins [28]. The DNA in the NETs can be detected by TLR receptors on pDCs leading to production of type I interferons. We therefore hypothesized that production of NETs might play a role in the excess pathology seen in *Apoe*^*-/-*^ mice following infection with Mtb. To block NET formation, we treated *Apoe*^*-/-*^ HC mice with GSK484 which has been demonstrated to block production of NETs by human and murine neutrophils by inhibiting PAD4, an enzyme required for citrullination of histones [43–45]. Treatment with GSK484 reduced the levels of NETs in the lung as measured by the presence of citrullinated histone H3 (Fig 4A and 4B) and decreased the expression of *Ifnb1* without decreasing the numbers of neutrophils or other immune cell types in the lung (Figs 4C, 4D and S6). Strikingly, inhibition of NET formation reduced the bacterial burden in the lungs of *Apoe*^*-/-*^ HC to approximately that measured in B6 mice (Fig 4E).

To explore the potential clinical efficacy of blocking NET formation, we infected *Apoe*^*-/-*^ HC mice with ~50 CFU of H37Rv and treated them with GSK484 daily starting at day 7 or 14 PI until a pre-specified endpoint of 40 days PI. Treatment with GSK484 significantly decreased mortality compared to controls (Figs 4F and S7). While deletion of *Padi4* has been shown to affect expression of MHC II on tumor associated macrophages [46], we did not measure any change in MHCII expression in monocyte-derived macrophages following GSK484 administration (Fig 4G).

To determine the generalizability of these findings, we tested the effect of blocking NET formation in a different mouse strain/ bacterial strain combination. C3HeB/FeJ (C3H) mice are highly susceptible to Mtb and the pathology in these mice has been shown to be driven, at least in part, by excess neutrophil recruitment, particularly when infected with the hypervirulent SA161 strain of Mtb [16,47]. Treatment of SA161-infected C3H mice with GSK484 decreased the bacterial burden by approximately 8-fold (Fig 4H). In contrast, blocking PAD4-induced NET formation with GSK484 in B6 mice did not affect the pulmonary bacterial burden or decrease the numbers of neutrophils or monocyte-derived macrophages (Fig 4I and 4J). These results are concordant with a minimal role for neutrophils in the pathology of Mtb infection in B6 mice [15] and suggest that GSK484 does not have any significant direct anti-mycobacterial activity under the *in vivo* conditions tested.

### Neutrophils in *Apoe*^*-/-*^ HC mice are biased towards an N2 phenotype

**Fig 5:**
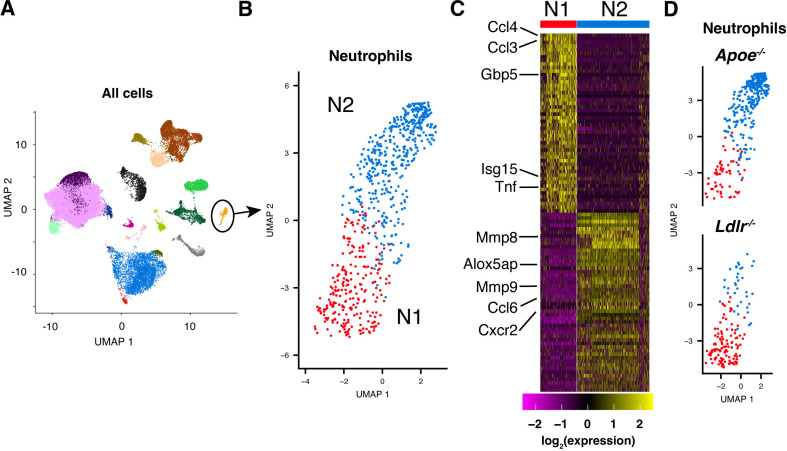
Neutrophils in *Apoe*^*-/-*^ HC mice have a distinct polarization state. (**A**) UMAP plot of expression measurements from single-cell RNA-seq analysis of pulmonary immune cells isolated from B6, *Apoe*^*-/-*^, and *Ldlr*^*-/-*^ HC mice at day 14 PI with ~50 CFU Mtb H37Rv (See Methods.). The neutrophil population, identified by comparison with the ImmGen database of transcriptional profiles (https://www.immgen.org) and confirmed by examining expression of *Ly6g* and *S100a8*, is shown in orange and circled. (**B**) UMAP plot of re-clustered expression measurements for the neutrophil population shown in (A). (**C**) Heatmap of row-normalized expression measures for the top 100 genes that distinguish the clusters labeled N1 and N2 in (B). (**D**) UMAP plot of neutrophils from *Apoe*^*-/-*^ and *Ldlr*^*-/-*^ HC mice at day 14 PI. The relative sizes of the N1 and N2 clusters are *Apoe*^*-/-*^ log_2_(N2/N1) = 2.1 ± 1.5 and *Ldlr*^*-/-*^ log_2_(N2/N1) = -1.8 ± 0.3 (mean ± SEM for 3 replicates). See S10 Fig for gating strategy.

In several of the experiments described above, the intervention improved the outcome of the mice without affecting the number of neutrophils recruited to the lung. This suggested that the state of the neutrophil when it encounters the Mtb-infected lung may be a critical determinant of disease outcome. To investigate this hypothesis, we examined the transcriptional profiles of *Apoe*^*-/-*^, *Ldlr*^*-/-*^, and B6 pulmonary neutrophils at an early time point prior to the divergence in the bacterial burden. We isolated intrapulmonary cells (defined by lack of labelling by an anti-CD45 antibody administered intravenously immediately prior to sacrifice) at day 14 following aerosol challenge with ~50 CFU of Mtb H37Rv and measured their transcriptomes by single-cell RNA-seq (Fig 5A). When the neutrophil population was isolated and re-clustered, it separated into two distinct populations that were distinguished by expression of numerous genes that correlate with the N1 and N2 phenotypes, as previously described for TANs [31–33], including *Tnf*, *Ccl3*, and *Ccl4* (N1) and *Mmp8*, *Mmp9*, and *Ccl6* (N2) (Fig 5B and 5C). At day 14, the N2 population was significantly larger in the highly susceptible *Apoe*^*-/-*^ HC mice compared to the relatively protected *Ldlr*^*-/-*^ HC mice (Fig 5D).

**Fig 6:**
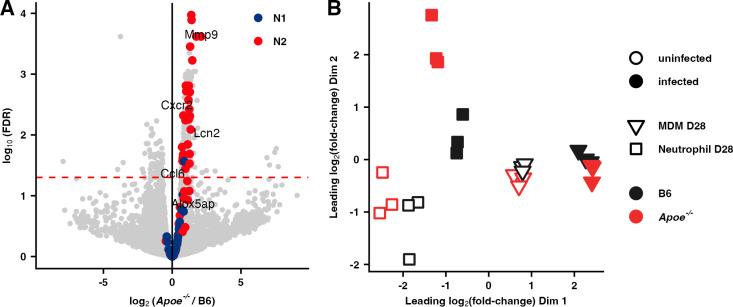
Transcriptional analysis of macrophages and neutrophils isolated from B6:*Apoe*^*-/-*^ mixed bone marrow chimeric mice. (**A**) Volcano plot depicting differential expression between *Apoe*^*-/-*^ and B6 bystander (uninfected) neutrophils isolated from B6:*Apoe*^*-/-*^ mixed bone marrow chimeric mice, maintained on a normal diet, 28 days following infection with ~50 CFU H37Rv. Genes that are most characteristic of N1 and N2 neutrophils in Mtb-infected mice on a HC diet as determined by single-cell RNA-seq analysis are colored (See Fig 5C). Dashed line indicates FDR = 0.05. (n = 3 mice/group) (**B**) Multidimensional scaling (MDS) plot^65^ of gene expression in alveolar macrophages (AM), monocyte-derived macrophages (MDM), and neutrophils isolated by cell sorting from B6:*Apoe*^*-/-*^ mixed bone marrow chimeric mice at Day 28 following infection with ~50 CFU of Mtb H37Rv expressing mCherry^66^. The top 500 genes with the largest standard deviations across samples were used to generate the plot. Open (filled) shapes indicate uninfected (infected) cells, shapes indicate cell types (△ = MDM, □ = Neutrophil), and colors indicate genotype (red = *Apoe*^*-/-*^, black = B6). Distances on the plot represent the leading log2-fold-changes, which are defined as the root-mean-square average of the top largest log2-fold-changes between each pair of samples. (n = 3 mice/group) See S10 Fig for gating strategy.

This skewing to an N2 phenotype was also observable in *Apoe*^*-/-*^ neutrophils isolated from B6:*Apoe*^*-/-*^ mixed bone-marrow chimeric mice infected with Mtb for 28 days (Fig 6A). Surprisingly, while we measured genotype-specific expression differences between neutrophils in this system, the expression profiles of monocyte-derived macrophages, both infected and uninfected, were quite similar (Fig 6B). A recent paper suggested that APOE, secreted from prostate cancer cells, can bind to TREM2 on neutrophils and drive them towards a senescent phenotype that promotes tumor progression [48]. Based on the fact that we find that the distinct transcriptional profile of *Apoe*^-/-^ neutrophils is preserved in B6:*Apoe*^-/-^ mixed bone-marrow chimeras on a B6 background (Fig 6A), we do not believe that this mechanism contributes to the pulmonary neutrophil polarization we observe in *Apoe*^-/-^ HC mice. In addition, this approach enabled us to examine the effect of deleting APOE on the bacterial burden per infected cell. We do not observe a significant difference in burden in either neutrophils or monocyte-derived macrophages between genotypes (S8 Fig).

## Discussion

Patients with severe tuberculosis, characterized by the development of cavitary lesions, are prone to prolonged culture positivity and increased risk of relapse leading to a higher chance of developing MDR TB and to lasting lung damage [49–51]. We have used the *Apoe*^-/-^ HC mouse which is hypersusceptible to infection with Mtb and which recapitulates several aspects of severe TB in humans, including the development of neutrophil-rich necrotic lesions [12], to identify immune mechanisms that are dysregulated during severe disease. Numerous recent studies have highlighted the correlation between high levels of neutrophils in the lung and poor outcome of TB and used depletion strategies to suggest a causal connection [16–18]. Depletion of neutrophils is unlikely to be a viable clinical treatment and in fact neutropenia is a well-known risk factor for severe disease and death following infection with multiple pathogens. An ideal host directed therapy (HDT) would be highly specific, targeting only those aspects of the immune response that are dysregulated in severe disease while preserving a robust host defense against other infections.

The specific neutrophil functions that lead to loss of control of Mtb growth or to severe pathology have not been well defined. Several groups have identified a correlation between increased NET formation and susceptibility to tuberculosis in mice [18,19,22], and in humans with tuberculosis, NET formation has been associated with necrotic granulomas that lead to cavitary lesions [19] which predispose patients to prolonged culture positivity, increased risk of relapse, and lasting lung damage [49–51]. Our experiments indicate that NET formation is a significant contributor to poor control of Mtb infection during severe disease and suggest that specifically blocking this process with GSK484 can improve outcomes. These results are consistent with a recent study that investigated the role of type I interferon in promoting NETosis and showed that using a different PAD4 inhibitor also improved the outcome of TB in mice [22]. Unlike neutrophil depletion strategies, blocking the formation of NETs is not broadly immunosuppressive [52,53] and in fact, in a model of polymicrobial sepsis, has been shown to offer a survival benefit [54].

Expression of type I interferon stimulated genes (ISGs) is elevated in the serum of humans with active TB (see [21] and references therein) and ISG-rich gene expression signatures correlate with and often predict aspects of TB disease including progression from latency to active disease [55] and outcome of treatment [56]. We found that type I interferon contributes to the severe disease phenotype in *Apoe*^*-/-*^ HC mice which is consistent with prior studies showing that type I interferon contributes to poor outcome following Mtb-infection at least in part by driving neutrophil activation and promoting NET formation [19]. However, in some murine models of severe TB such as *Nos2*^-/-^ and *Acod1*^-/-^, the susceptibility is independent of type I interferon signaling despite the fact that dysregulated neutrophil responses also play a detrimental role in these strains [16,17,41], suggesting that the impact on disease severity may depend on the detailed mechanisms by which susceptibility arises in each background.

It is increasingly appreciated that mature neutrophils in the periphery can be polarized towards different states. In cancer, polarization of TANs has been shown to play an important role in the immune response: N1 neutrophils are considered inflammatory, express high levels of *Tnf* and restrain tumorigenesis through cytotoxicity and enhancement of anti-tumor responses while N2 neutrophils, which strongly express genes such as *Mmp8* and *Mmp9* are thought to stimulate tumor growth by promoting remodeling of extracellular matrix, enhancing angiogenesis, and inhibiting cytotoxic T-cell responses [31–33]. While this paradigm has not been well studied in the context of infectious diseases, it is tempting to speculate that in Mtb infection remodeling responses might promote destruction of extracellular matrix which could bias toward necrotic cavity formation and long-term tissue damage. Furthermore, some studies in cancer models suggest that N2 TANs are more prone to NETosis and that, reciprocally, NETosis skews neutrophils in the tumor microenvironment to an N2 phenotype, possibly by MMP9 activation of latent TGFb in the NET [57–59]. Consistent with this hypothesis we have identified a distinct neutrophil transcriptional profile in *Apoe*^-/-^ mice that are highly susceptible to infection with Mtb which is similar to that described in N2 TANs, while the transcriptional profile of neutrophils from *Ldlr*^*-/-*^ mice that are more resistant to Mtb is similar to that described in N1 TANs. A recent study examined neutrophils isolated from TB patients who were stratified by the magnitude of their lymphocyte proliferative responses and found that surface markers characteristic of an N1 phenotype were more highly expressed on neutrophils from patients with strong responses while surface markers characteristic of N2 neutrophils were more highly expressed on patients with weaker responses [34]. Due to differences in the reported duration of symptoms prior to hospitalization between high and low responders [34], additional studies earlier in the course of disease, or even prospectively, will be required to establish a causal link between N1/N2 polarization and modulation of lymphocyte responses. In patients with necrotic granulomatous lesions, adjunctive HDTs that block or modify inflammatory mechanisms that lead to matrix destruction and lung injury, and that enhance antimicrobial drug penetration and action would be particularly useful. Thus, determining whether skewing of neutrophils towards a particular state drives poor outcomes in TB would enable leveraging the ongoing work in cancer to manipulate these states for treating TB and other chronic infectious diseases.

Based on animal studies, blockade of NETs is postulated to be beneficial in multiple conditions including atherosclerotic vascular disease, arthritis, and several types of cancer [44,53,60–62]. While no PAD4 inhibitors are currently FDA-approved, several are in pre-clinical development by multiple companies [53,62].

## Materials and methods

### Ethics statement

All experiments were approved by the Institutional Animal Care and Use Committee at Seattle Children’s Research Institute and then performed in compliance with the relevant protocols.

### Mice

WT (C57BL6/J, JAX:000664, RRID:IMSR_JAX:000664), *Apoe*^*-/-*^ (B6.129P2-Apoe^tm1unc/J^, JAX:002052, RRID:IMSR_JAX:002052), *Ldlr*^*-/-*^ (B6.129S7-Ldlr^tm1Her/J^, JAX:002207, RRID:IMSR_JAX:002207), WT CD45.1 (B6.SJL-Ptprc^a^ Pepc^b^/BoyJ, JAX #002014, RRID:IMSR_JAX:002014), C3H (C3HeB/FeJ, JAX#000658), OT-I (C57BlV6-Tg(TcraTcrb)1100Mjb/J, JAX#003831, RRID:IMSR_JAX:003831), OT-II (B6.Cg-Tg(TcraTcrb)425Cbn/J, JAX#004194, RRID:IMSR_JAX:004194), and ESAT-6 TCR Tg (C7) (JAX035728, RRID:IMSR_JAX:035728) strains of *Mus musculus* were obtained from the Jackson Laboratories (Bar Harbor, ME). OT-I and OT-II mice were crossed onto the CD45.1 mice. All KO mouse experiments used only homozygous animals. All mice were housed in group housing not exceeding 5 animals per cage and maintained in specific pathogen–free conditions at the Seattle Children’s Research Institute (SCRI). Mice were maintained on standard chow (PicoLab Rodent Diet 20, LabDiet). In experiments where mice were fed a HC diet the animals were switched to diet D12109C (Research Diets) 14 days prior to infection and maintained on this diet throughout the remainder of the experiment. Healthy 8- to 14-week-old mice without any previous procedure history were used for all experiments and age and sex matched within each experiment. Our study examined male and female animals, and the findings were similar for both sexes.

### T cell priming assays

OVA-specific CD4+ or CD8 + T cells or TB-specific CD4 + T cells were prepared from spleen and lymph nodes of OT-II, OT-I, or C7 TCR transgenic mice by negative selection using the CD4+ or CD8 + T Cell Isolation Kit (Miltenyi Biotec., #130-104-454, #130-104-075) according to the manufacturer’s instructions. For T cell proliferation assays, purified T cells were labeled with 2 μM CFSE (ThermoFisher, #C34554) before transfer. 10^6^ purified OT-II CD4 + , OT-I CD8 + , or C7 CD4 + T cells were adoptively transferred into mice by retro-orbital injection. 24 hours later, mice were infected either IN (for BCG-OVA) or ID (for H37Rv) with the indicated doses of live BCG-OVA or H37Rv. Draining lymph nodes were harvested 4 days (mLNs for BCG-OVA) or 5 days (cLNs for H37Rv) PI, and single-cell suspensions were prepared, stained and fixed, and then analyzed on a LSRII or A5 flow cytometer (BD Bioscience).

### T cell function assays

Single-cell suspensions were made from murine lung and were stimulated with TB10.4 (IMYNYPAM) peptide (5 μg/mL final concentration) for 4–6 hours in complete RPMI 1640 media in the presence of 1 μg/mL anti-CD28, anti-CD49d, and Brefeldin A (10 μg/mL) at 37 °C with 5% CO_2_. Cells were washed and surface stained for 30 minutes in the dark at 4 °C, then fixed and permeabilized using BD Cytofix/Cytoperm Fixation/Permeabilization kit (Cat # 555028) for intracellular cytokine staining.

### Mouse Mtb aerosol infection

For standard-dose (~50 CFU) infections, mice were enclosed in an aerosol infection chamber (Glas-Col) and frozen stocks of bacteria were thawed, diluted 1:75 in 0.01% Tween-80 in water, and placed inside the associated nebulizer. To determine the infectious dose, three mice in each infection were sacrificed after the aerosolization was complete. The whole lung was homogenized in 0.05% Tween-80 in PBS with a gentleMACS Tissue Dissociator (Miltenyi Biotec) and serial dilutions were plated onto 7H10 plates for CFU enumeration, as described previously [63]. All infections used the H37Rv strain of Mtb unless otherwise indicated. The SA161 strain of Mtb was provided by Ian Orme (Colorado State University).

#### Neutrophil depletion.

Mice were fed a HC diet for 14 days prior to infection. Mice received either 200 μg of anti-Ly6g antibody (Anti-mouse Ly6G, BioXCell, Cat #BP0075–1, RRID:AB_1107721) or of isotype control (Rat IgG2a, BioXCell, Cat#BP0089, RRID: AB_1107769) via IP injection starting 2 days prior to infection and then every 3 days thereafter for the duration of the experiment.

#### pDC depletion.

Mice were fed a HC diet for 14 days prior to infection. At days -3 and -1 prior to infection and then every 5 days thereafter throughout the experiment, mice received 0.25 mg of anti-PDCA1 antibody (Anti-mouse CD317, BioXCell, Cat #BE0311, RRID:AB_2736991) or isotype control antibody (Rat IgG2b, BioXCell, Cat#BE0090, RRID:AB_1107780) via IP injection.

#### Blocking IFNAR1.

Mice were fed a HC diet for 14 days prior to infection. Mice received either 0.5 mg of anti-IFNAR1 antibody (Leinco Technologies, Clone MAR1-5A3, RRID:AB_2830518) or of isotype control (Leinco Technologies, Cat #G737, Clone GIR-208, RRID:AB_2830302) via IP injection starting 2 days prior to infection and then every 3 days thereafter for the duration of the experiment.

#### Inhibiting PAD4.

Mice were fed a HC diet for 14 days prior to infection. Mice received an IP injection of 0.2 mg of GSK484 (MedChem Express, HY-100514) or PBS with 4% DMSO daily starting 7 days post-infection for the duration of the experiment.

### Bone marrow transplantation

Bone marrow was harvested by flushing the femurs of the donor mice. B6 CD45.1 mice (B6.SJL-Ptprc^a^ Pepc^b^/BoyJ) were irradiated with two doses of 500 rads using an X-Rad 320 irradiator, then reconstituted with 10^6^ bone marrow cells as, 10^6^ cells from 1:1 mix of *Apoe*^*-/-*^ (CD45.2) and B6 CD451/2 (C57BL/6 x B6.SJL-Ptprc^a^ Pepc^b^/BoyJ) bone marrow. Mice were allowed to recover for 8 weeks and then placed on HC diet for 14 days prior to infection. Engraftment was confirmed by flow cytometry.

### Cell sorting and flow cytometry

Samples for flow cytometry and cell sorting were prepared as described previously [63]. Significant details are presented here.

#### Isolation of single-cell suspensions from lung.

For T cell experiments, at the indicated times post-infection, mice were anesthetized with isoflurane and administered 1 μg APC-labeled anti-CD45 antibody intravenously to distinguish cells in the circulation (IV+) from those in the lung parenchyma (IV-). Five minutes later, mice were euthanized by CO_2_ asphyxiation, lungs harvested in HEPES buffer containing Liberase Blendzyme 3 (70 μg/mL; Roche, #05401020001) and DNaseI (30 μg/ml; Sigma-Aldrich, #10104159001), and lightly homogenized using a gentleMacs dissociator (Miltenyi Biotec). The lightly homogenized lungs were then incubated for 30 min at 37 °C and then homogenized a second time using the gentleMacs. The homogenates were filtered through a 70 μm cell strainer, pelleted for RBC lysis with ACK lysing buffer (ThermoFisher, #A1049201), and resuspended in FACS buffer (PBS containing 2.5% FBS).

#### Flow cytometry analysis and antibodies.

For surface staining, cells were suspended in 1X PBS (pH 7.4) containing 0.01% NaN_3_ and 1% fetal bovine serum and blocked with anti-CD16/32 (2.4G2, BD Bioscience), then labeled at 4 °C for 30 minutes in the dark. For intracellular cytokine detection, cells were surface stained and fixed, and then permeabilized. Cell viability was assessed using Live/Dead fixable Aqua or Blue dye (ThermoFisher, #L34966, #L23105). Stained cells were analyzed on a BD LSR II or A5 flow cytometer (BD Bioscience). Samples for flow cytometry were fixed in 2% paraformaldehyde solution in PBS and analyzed using a LSRII or A5 flow cytometer (BD Biosciences) and FlowJo software (Tree Star, Inc.).

The following reagents were used for flow cytometry analysis:

BST2: PE anti-mouse CD317 (BST2, PDCA-1) Antibody (927) (BioLegend, Cat # 127010, RRID:AB_1953285)

CD4: BD Horizon BUV496 Rat Anti-Mouse CD4 (GK1.5) (BD Biosciences, Cat # 612952, RRID:AB_2813886)

CD8a: BD Horizon BUV395 Rat Anti-Mouse CD8a (53-6.7) (BD Biosciences, Cat # 563786, RRID:AB_2732919)

CD11b: Brilliant Violet 570 anti-mouse/human CD11b Antibody (M1/70) (BioLegend, Cat # 101233, RRID:AB_10896949)

CD11c: Brilliant Violet 605 anti-mouse CD11c Antibody (N418) (BioLegend, Cat # 117333, RRID:AB_11204262)

CD11c: APC/Fire 750 anti-mouse CD11c Antibody (N418) (BioLegend, Cat # 117352, RRID_AB_2572124)

CD16/32: TruStain FcX (anti-mouse CD16/32) Antibody (BioLegend, Cat # 101320, RRID:AB_1574973)

CD19: BD OptiBuild BUV563 Rat Anti-Mouse CD19 (1D3) (BD Biosciences, Cat # 749028, RRID:AB_2873425)

CD45: FITC anti-mouse CD45 Antibody (30-F11) (BioLegend, Cat # 103108, RRID: AB_312973)

CD45: PerCP/Cyanine5.5 anti-mouse/human CD45R/B220 Antibody (RA3-6B2) (BioLegend, Cat # 103236, RRID:AB_893354)

CD45: APC anti-mouse CD45 Antibody (30-F11) (BioLegend, Cat # 103112, RRID:AB_312977)

CD64: PE/Cyanine7 anti-mouse CD64 (FcγRI) Antibody (X54-5/7.1) (BioLegend, Cat # 139314, RRID:AB_2563904)

Ly6c: BD OptiBuild BUV805 Rat Anti-Mouse Ly-6C (HK1.4.rMAb) (BD Biosciences, Cat # 755202, RRID:AB_11204262)

Ly6c: Brilliant Violet 785 anti-mouse Ly-6C Antibody (HK1.4) (BioLegend, Cat # 128041, RRID:AB_2565852)

Ly6g: Brilliant Violet 711 anti-mouse Ly-6G Antibody (1A8) (BioLegend, Cat # 127643, RRID:AB_2565971)

MHCII: BD OptiBuild BUV615 Rat Anti-Mouse I-A/I-E (M5/114.15.2) (BD Biosciences, Cat # 751570, RRID:AB_2875565)

MHCII: Brilliant Violet 650 anti-mouse I-A/I-E Antibody (M5/114.15.2) (BioLegend, Cat # 107641, RRID:AB_2565975)

NK1.1: Brilliant Violet 785 anti-mouse NK-1.1 Antibody (PK136) (BioLegend, Cat # 108749, RRID:AB_2564303)

SiglecF: BD Horizon BV421 Rat Anti-Mouse Siglec-F (E50-2440)(BD Biosciences, Cat # 562681, RRID:AB_2722581)

SiglecF: PE/Dazzle 594 anti-mouse CD170 (Siglec-F) Antibody (S17007L) (BioLegend, Cat # 155530, RRID:AB_2890716)

TCRβ: BUV737 Hamster Anti-Mouse TCR β Chain (H57-597) (BD Biosciences, Cat # 612821, RRID:AB_2870145)

TNF: APC anti-mouse TNF-α Antibody (BioLegend, Cat # 506308)

Live/Dead discrimination: LIVE/DEAD Fixable Aqua Dead Cell Stain Kit, for 405 nm excitation (ThermoFisher, Cat # L34966); LIVE/DEAD Fixable Blue Dead Cell Stain Kit, for UV excitation (ThermoFisher, Cat # L23105)

Tetramers: Anti-MHC class I TB10.4 tetramer (NIH Tetramer Core Facility, sequence: IMYNYPAM)

#### Cell sorting.

Lungs were dissociated as described above and resuspended in RPMI (Gibco, #11875093) for labeling. Cell sorting was performed on a FACS Aria II (BD Biosciences). Sorted cells were collected in complete media, pelleted, resuspended in TRIzol, and frozen at -80°C overnight prior to RNA isolation.

### Confocal microscopy

Lungs were dissected and incubated in BD Cytofix diluted 1:3 with PBS for 24 hours at 4 °C. Lungs were then washed two times in PBS, incubated in 30% sucrose for 24 hours at 4 °C, embedded in OCT, and frozen in a dry ice slurry with 100% ethanol. 20 μm sections were cut using a CM1950 cryostat (Leica) and placed on charged slides. Sections were rehydrated with 0.1 M TRIS for 10 minutes at room temperature, incubated for 1 hour at room temperature with blocking buffer (0.1 M TRIS with 1% normal mouse serum, 1% bovine serum albumin, and 0.3% Triton X100), and then incubated overnight at room temperature with fluorescently conjugated antibodies or DNA dyes (Nucspot Nuclear Stains 750/780, Biotium, #41038; Mycobacterium tuberculosis purified protein derivative (PPD-Alexa488), Abcam, Cat # ab20962, RRID:AB_445945; Anti-mouse Histone H3CitAbcamCat # ab281584). Following labeling, slides were washed with 0.1 M TRIS for 30 minutes and sections sealed with coverslips and Fluoromount G mounting media (Southern Biotech, 0100–01). Images were acquired on a Leica Stellaris8 confocal microscope at room temperature using a 63X/NA1.20 HC PL APO water-coupled objective. For visual clarity, thresholds were applied to the displayed channel intensities using ImageJ with identical settings applied across experimental groups. To quantify the level of citrullinated histone H3 (Cit-H3) signal in each section, discrete lesions were identified visually based on purified protein derivative (PPD) antibody labeling and the fluorescent intensity of Cit-H3 labeling measured in 5 independent regions within the lesion. Background fluorescence was estimated using a similar analysis of unlabeled tissue sections.

### Gene expression analysis

#### Real-time PCR of lung tissue.

The right superior lobe of the lungs was placed in TRIzol (Invitrogen, 15596018) and isolated using two sequential chloroform extractions, Glycoblue carrier (Invitrogen, AM9515), isopropanol precipitation, and washes with 75% ethanol. cDNA was synthesized using the RNA to cDNA EcoDry kit (Takara #693543) Expression of *Ifnb1* was measured using TaqMan primer probes (ThermoFisher, Mm00439552_s1), TaqMan Fast Universal PCR Master Mix (ThermoFisher, #4364103), and a Quant Studio 5 RT-qPCR detection system (ThermoFisher). Measurements were normalized to expression of *Eef1a1* expression in individual samples (Integrated DNA technologies - *Eef1a1* forward primer for custom TaqMan assay: 5’ GCAAAAACGACCCACCAATG 3’, *Eef1a1* reverse primer for custom TaqMan assay: 5’ GGCCTGGATGGTTCAGGATA 3’, *Eef1a1* probe for custom TaqMan assay: 5’/56-FAM/CACCTGAGCAGTGAAGCCAG/36-TAMSp/3’).

#### Bulk RNA-seq.

RNA isolation was performed using TRIzol, two sequential chloroform extractions, Glycoblue carrier (Invitrogen, AM9515), 100% isopropanol precipitation, two washes with 70% ethanol, and final resuspension in RNase free water. RNA was quantified with the Bioanalyzer RNA 6000 Pico Kit (Agilent, 5067–1513). cDNA libraries were constructed using the SMARTer Stranded Total RNA - Pico Input Mammalian Kit (TaKaRa, 634411) following the manufacturer’s instructions. Libraries were amplified and then sequenced on an Illumina NovaSeq 6000 (150 bp paired-end). The read pairs were aligned to the mouse genome (mm10) using the gsnap aligner [64]. Concordantly mapping read pairs (∼20 million/ sample) that aligned uniquely were assigned to exons using the subRead program [65] and gene definitions from Ensembl Mus_Musculus GRCm38.78 coding and non-coding genes. Genes with low expression were filtered using the “filterByExpr” function in the edgeR package [66] from bioconductor.org. Differential expression was calculated using the “edgeR” package and false discovery rate computed with the Benjamini-Hochberg algorithm. Data are available in GEO (GSE272446).

#### Single-cell RNAseq.

Libraries were prepared using the Next GEM Single Cell 3′ Reagent Kits v3.1 (Dual Index) (10X Genomics, PN-1000268) following the manufacturer’s instructions. Raw sequencing data were aligned to the mouse genome (mm10) and UMI counts determined using the Cell Ranger pipeline (10X Genomics). Data processing, integration, and analysis was performed with Seurat v.3 [67]. Droplets containing less than 200 detected genes, more than 4000 detected genes (doublet discrimination), or more than 5% mitochondrial reads were discarded. Genes expressed by less than 3 cells across all samples were removed. Unbiased annotation of clusters using the Immgen databasez [68] as a reference was performed with the “SingleR” package [69]. Data visualization was performed with the “Seurat”, “tidyverse”, “cowplot”, and “viridis” R packages.

Data are available in GEO (GSE272772).

### Serum cholesterol analysis

Total cholesterol, HDL, LDL, and triglyceride levels were measured using the Rodent Lipid Panel by IDEXX BioAnalytics (Test Code 6290).

### Statistical analysis

Statistical analysis was performed in R (v4.4.0). Definitions of center and dispersion are indicated in the figure captions. Measurements from individual replicates are indicated with points and unless otherwise noted indicate individual mice. Statistical significance was determined using the two-sided Student’s t-test allowing for unequal variances. Statistical significance of differences in measurements of bacterial burden by CFU analysis was assessed using the Wilcox rank-sum test. Significance of survival experiments was assessed using the log-rank (Mantel-Haenszel) test to test for a difference between Kaplan-Meier survival curves.

## Supporting information

S1 Fig *Apoe*^*-/-*^ HC mice are highly susceptible to infection with Mtb. (**A**) Female mice of the indicated genotypes were fed either normal food or high-cholesterol food for two weeks and then infected with ~50 CFU Mtb H37Rv and maintained on their pre-infection diet. (n = 8–9 mice/group) (B) Serum cholesterol profiles at day 7 following infection of the indicated genotypes of mice fed HC food and infected with Mtb H37Rv as in (A). HDL = high-density lipoproteins, LDL = low-density lipoproteins. (n = 3 mice/group) (**C**) Mice of the indicated genotypes were fed normal chow and then infected with ~50 CFU Mtb H37Rv. Bacterial burden in the lung was measured at day 28 PI by CFU counting. (n = 5–7 mice/group) Bars/lines indicate mean; error bars indicate SEM. Significance analysis was performed using the Mantel-Haenszel test (A) or the Wilcox rank-sum test (C).
(TIF)

S2 Fig Pulmonary cellularity in B6, *Apoe*^*-/-*^, and *Ldlr*^*-/-*^ mice following Mtb infection. Mice of the indicated genotypes on a HC diet were infected with ~50 Mtb H37Rv and the total number of each cell type in the lung determined by flow cytometry. Uninfected B6 mice on a normal diet were processed similarly for comparison. Bars indicate mean; error bars indicate SEM. See S10 Fig. for gating strategy. Significance analysis for a difference between *Apoe*^*-/-*^ and *Ldlr*^*-/-*^ mice was performed using the two-sided Student’s t-test allowing for unequal variances and the Benjamini-Hochberg correction for multiple comparisons. (n = 4–7 mice/group).
(TIF)

S3 Fig Elevated neutrophil levels in *Apoe*^*-/-*^ HC mice. (**A**) Mice of the indicated genotypes were maintained on normal chow or a HC diet for 14 days and the total numbers of neutrophils in lung was determined by flow cytometry. (n = 3–4 mice/group). (**B**) *Apoe*^*-/-*^ or B6 mice were placed on a HC diet for two weeks, infected with ~50 CFU H37Rv via aerosol, and maintained on the diet for the entire experiment. Total numbers of the indicated cell types in the lung at day 24 PI following the indicated treatments were measured by flow cytometry. Data are representative of 2 independent experiments. (n = 4–8 mice/group) Significance analysis for a difference between treated and control mice was performed using the two-sided Student’s t-test allowing for unequal variances and the Benjamini-Hochberg correction for multiple comparisons.
(TIF)

S4 Fig Pulmonary cellularity in *Apoe*^*-/-*^ HC mice following IFNAR blockade. *Apoe*^*-/-*^ or B6 mice were placed on a HC diet for two weeks, infected with ~50 CFU H37Rv via aerosol, and maintained on the diet for the entire experiment. Total numbers of the indicated cell types in the lung at day 21 PI following the indicated treatments were measured by flow cytometry. Significance analysis for a difference between treated and control mice was performed using the two-sided Student’s t-test allowing for unequal variances and the Benjamini-Hochberg correction for multiple comparisons. (n = 5–6 mice/group).
(TIF)

S5 Fig Pulmonary cellularity in *Apoe*^*-/-*^ HC mice following pDC depletion. *Apoe*^*-/-*^ or B6 mice were placed on a HC diet for two weeks, infected with ~50 CFU H37Rv via aerosol, and maintained on the diet for the entire experiment. Total numbers of the indicated cell types in the lung at day 28 PI following the indicated treatments were measured by flow cytometry. Significance analysis for a difference between treated and control mice was performed using the two-sided Student’s t-test allowing for unequal variances and the Benjamini-Hochberg correction for multiple comparisons. (n = 4–7 mice/group).
(TIF)

S6 Fig Pulmonary cellularity in *Apoe*^*-/-*^ HC mice following GSK484 treatment. *Apoe*^*-/-*^ or B6 mice were placed on a HC diet for two weeks, infected with ~50 CFU H37Rv via aerosol, and maintained on the diet for the entire experiment. Total numbers of the indicated cell types in the lung at day 28 PI following the indicated treatments were measured by flow cytometry. Significance analysis for a difference between treated and control mice was performed using the two-sided Student’s t-test allowing for unequal variances and the Benjamini-Hochberg correction for multiple comparisons. (n = 4–7 mice/group).
(TIF)

S7 Fig Survival analysis of *Apoe*^*-/-*^ HC mice following GSK484 treatment. *Apoe*^*-/-*^ HC mice were placed on a HC diet for two weeks, infected with ~50 CFU H37Rv via aerosol, maintained on the diet for the entire experiment, and treated with GSK484 or vehicle daily starting at day 7 PI. The fraction of mice surviving to day 40 is plotted. (n = 6 mice/group). Data presented is a replicate experiment for the data presented in Fig 4F.
(TIF)

S8 Fig Comparison of bacterial burden in *Apoe*^*-/-*^ or B6 innate immune cells in a mixed bone-marrow chimera following infection with Mtb. *Apoe*^*-/-*^:B6 mixed bone-marrow chimeric mice on a B6 background were infected via aerosol with ~50 CFU H37Rv. At day 28 PI, monocyte-derived macrophages (MDMs) and neutrophils were isolated by flow sorting. (**A**) Fraction of MDMs and neutrophils of the indicated genotypes. Blue bars indicate the fraction of circulating CD45 + cells of each genotype prior to infection. (**B**) Mean bacterial burden in MDMs or neutrophils of each genotype as determined by CFU plating of cells isolated in (A).
(TIF)

S9 Fig Gating strategies for T cell priming experiments. (**A**) *Expansion of adoptively transferred T cells in vivo:* The percentage of antigen-specific T cells dividing is computed relative to the total number of single T cells expressing CD45.1 (and CD8 or CD4 in the case of OT-II and OT-I T cells) (**B**) *Absolute counts of total T cells in the lung parenchyma:* Single CD45 + cells and counting beads are separated from debris by forward scatter and the absolute number of IV- cells computed relative to the known number of beads added. (**C**) *Fraction of antigen-specific T cells in the lung:* The percentage of TB10.4 tetramer+ T cells in the lung was defined relative to the number of single, CD45 + TCRb + CD8 + CD44 + T cells. (D) *Ex vivo T cell restimulation:* The fraction of CD8 T cells producing both IFNG and TNF was computed relative to the total number of single, live CD3 + CD8 + T cells.
(TIF)

S10 Fig Gating strategy for pulmonary cellularity, single-cell RNA-seq, and mixed bone-marrow chimera analysis. (**A**) Single, live, CD45 + cells were identified as indicated. (**B**) Prior to sacrifice, mice were injected with an PE-CD45.2 antibody to mark cells in the circulation. Following digestion of the lung to a single-cell suspension, single, live cells were gated on low Ter119 to exclude red blood cells and low PE-CD45.2 to define the population in the lung parenchyma. In addition, alveolar macrophages (AMs), were defined as SiglecF + CD11c+ cells, regardless of PE-CD45.2 status (IV labeling is unreliable for AMs due to high background autofluorescence.). These two populations were combined and sorted for analysis by single-cell RNA-seq. (**C**) Mixed bone marrow chimeric mice on a B6 background (B6.SJL-Ptprc^a^ Pepc^b^/BoyJ (CD45.1)) were generated by reconstitution with a 50:50 mixture of B6 (CD45.1.2) and *Apoe*^*-/-*^ (CD45.2) bone marrow. To isolate monocyte-derived macrophages (MDMs) and neutrophils single, CD45.2 + , CD19- cells were selected. SiglecF+ alveolar macrophages (AMs) were excluded and infected/uninfected (mCherry + /-) Ly6G+ neutrophils of each genotype isolated by CD45.1 level. Infected/uninfected (mCherry + /-) Ly6G- SiglecF- CD64 + CD11b + MDMs of each genotype were isolated by CD45.1 level.
(TIF)

S1 Data This file contains the data values for each plot in the main manuscript and supplementary information. (XLSX)
