# Supplementary figures and images for: APOE protects against severe infection with Mycobacterium tuberculosis by restraining production of neutrophil extracellular traps
Source: PLoS Pathog. 2025 Jun 16;21(6):e1013267. doi: 10.1371/journal.ppat.1013267 (PMC12201663; doi:10.1371/journal.ppat.1013267)

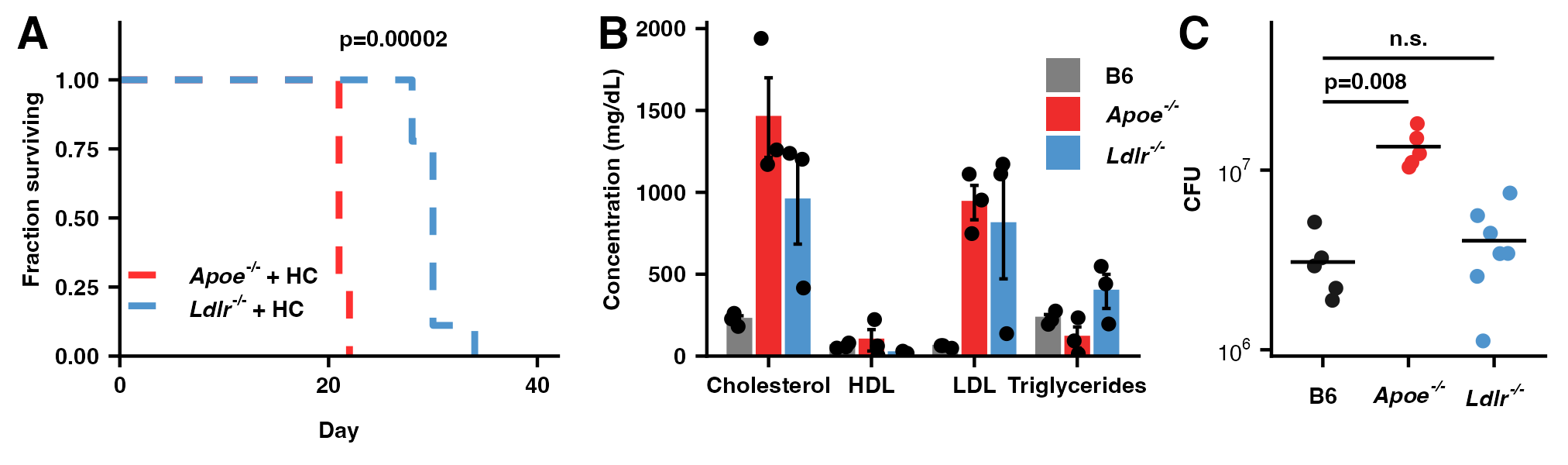

Supplement: S1 Fig — (A) Female mice of the indicated genotypes were fed either normal food or high-cholesterol food for two weeks and then infected with ~50 CFU Mtb H37Rv and maintained on their pre-infection diet. (n = 8–9 mice/group) (B) Serum cholesterol profiles at day 7 following infection of the indicated genotypes of mice fed HC food and infected with Mtb H37Rv as in (A). HDL = high-density lipoproteins, LDL = low-density lipoproteins. (n = 3 mice/group) (C) Mice of the indicated genotypes were fed normal chow and then infected with ~50 CFU Mtb H37Rv. Bacterial burden in the lung was measured at day 28 PI by CFU counting. (n = 5–7 mice/group) Bars/lines indicate mean; error bars indicate SEM. Significance analysis was performed using the Mantel-Haenszel test (A) or the Wilcox rank-sum test (C). (TIF) [file ppat.1013267.s001.tif]

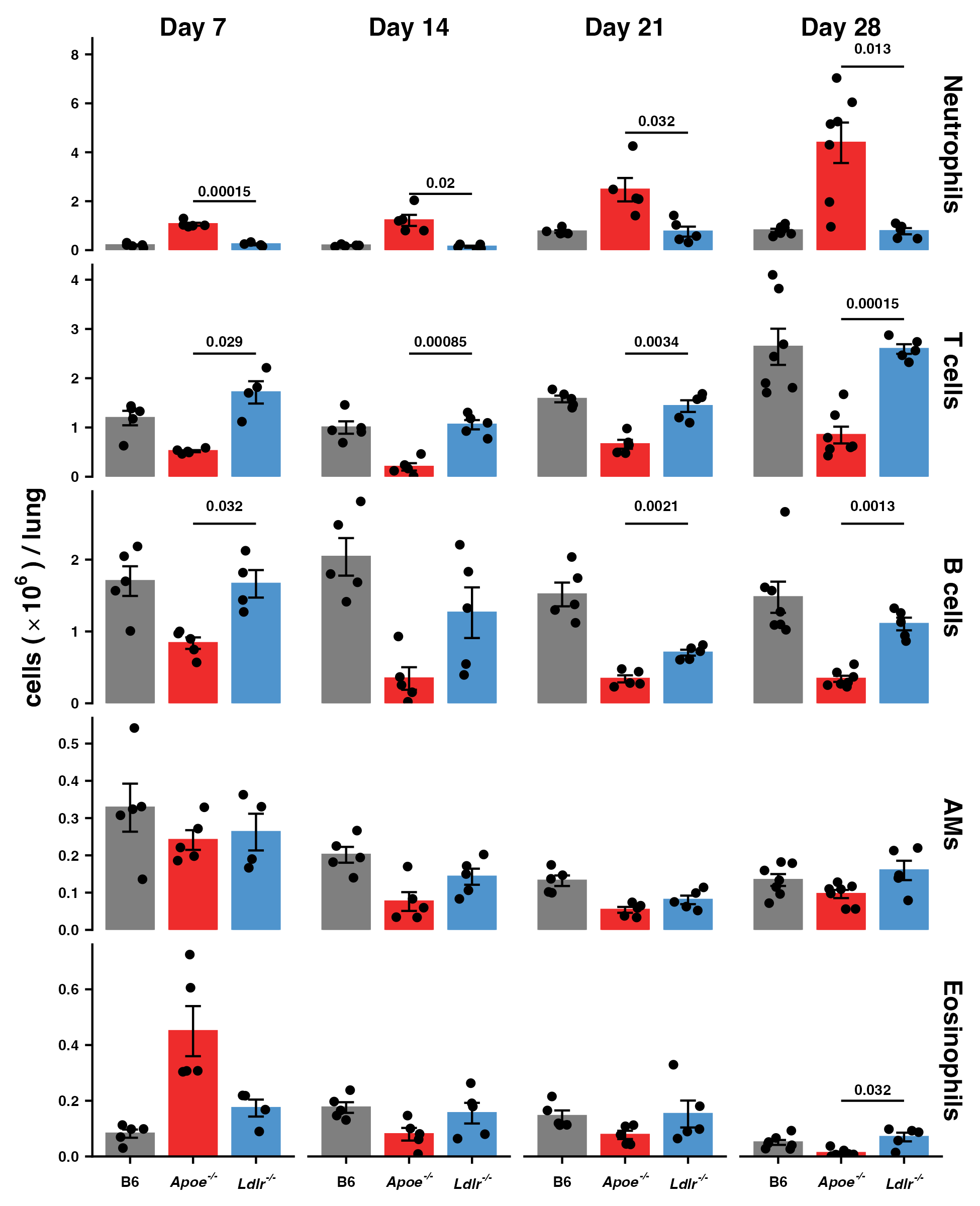

Supplement: S2 Fig — Mice of the indicated genotypes on a HC diet were infected with ~50 Mtb H37Rv and the total number of each cell type in the lung determined by flow cytometry. Uninfected B6 mice on a normal diet were processed similarly for comparison. Bars indicate mean; error bars indicate SEM. See S10 Fig. for gating strategy. Significance analysis for a difference between Apoe-/- and Ldlr-/- mice was performed using the two-sided Student’s t-test allowing for unequal variances and the Benjamini-Hochberg correction for multiple comparisons. (n = 4–7 mice/group). (TIF) [file ppat.1013267.s002.tif]

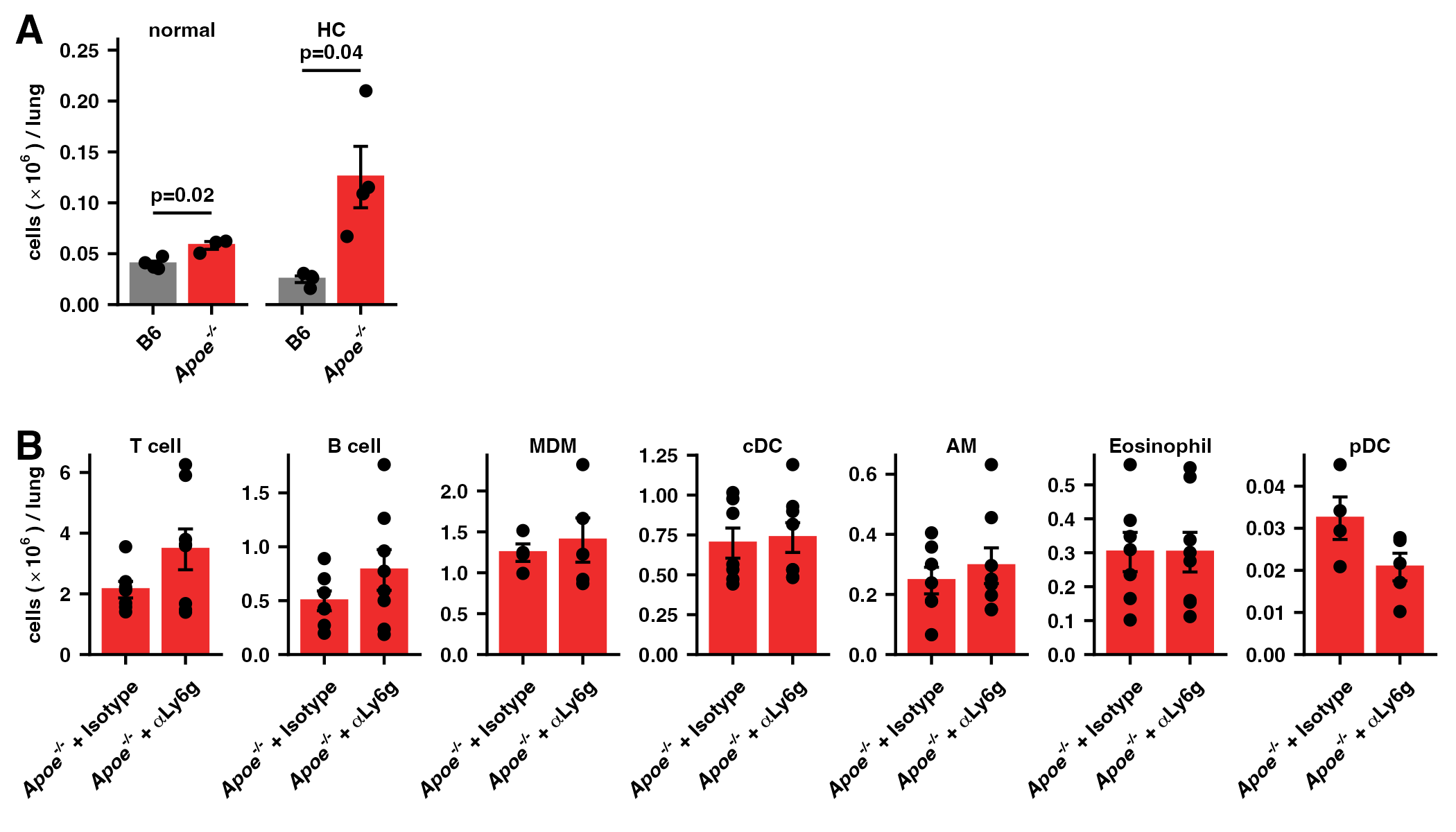

Supplement: S3 Fig — (A) Mice of the indicated genotypes were maintained on normal chow or a HC diet for 14 days and the total numbers of neutrophils in lung was determined by flow cytometry. (n = 3–4 mice/group). (B) Apoe-/- or B6 mice were placed on a HC diet for two weeks, infected with ~50 CFU H37Rv via aerosol, and maintained on the diet for the entire experiment. Total numbers of the indicated cell types in the lung at day 24 PI following the indicated treatments were measured by flow cytometry. Data are representative of 2 independent experiments. (n = 4–8 mice/group) Significance analysis for a difference between treated and control mice was performed using the two-sided Student’s t-test allowing for unequal variances and the Benjamini-Hochberg correction for multiple comparisons. (TIF) [file ppat.1013267.s003.tif]

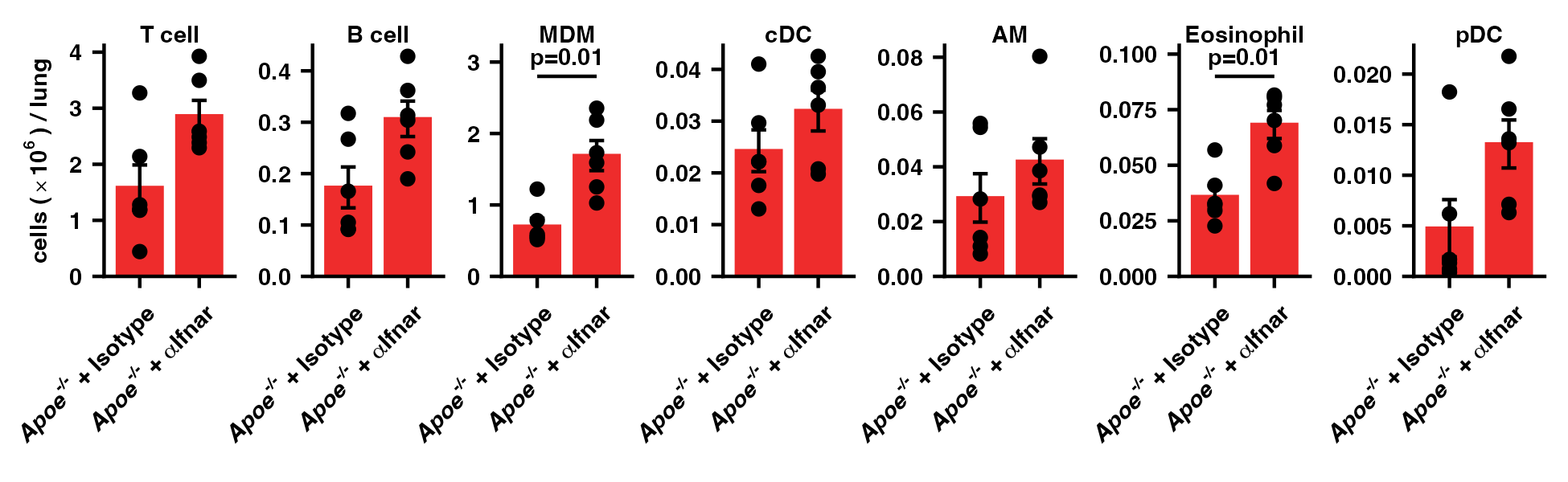

Supplement: S4 Fig — Apoe-/- or B6 mice were placed on a HC diet for two weeks, infected with ~50 CFU H37Rv via aerosol, and maintained on the diet for the entire experiment. Total numbers of the indicated cell types in the lung at day 21 PI following the indicated treatments were measured by flow cytometry. Significance analysis for a difference between treated and control mice was performed using the two-sided Student’s t-test allowing for unequal variances and the Benjamini-Hochberg correction for multiple comparisons. (n = 5–6 mice/group). (TIF) [file ppat.1013267.s004.tif]

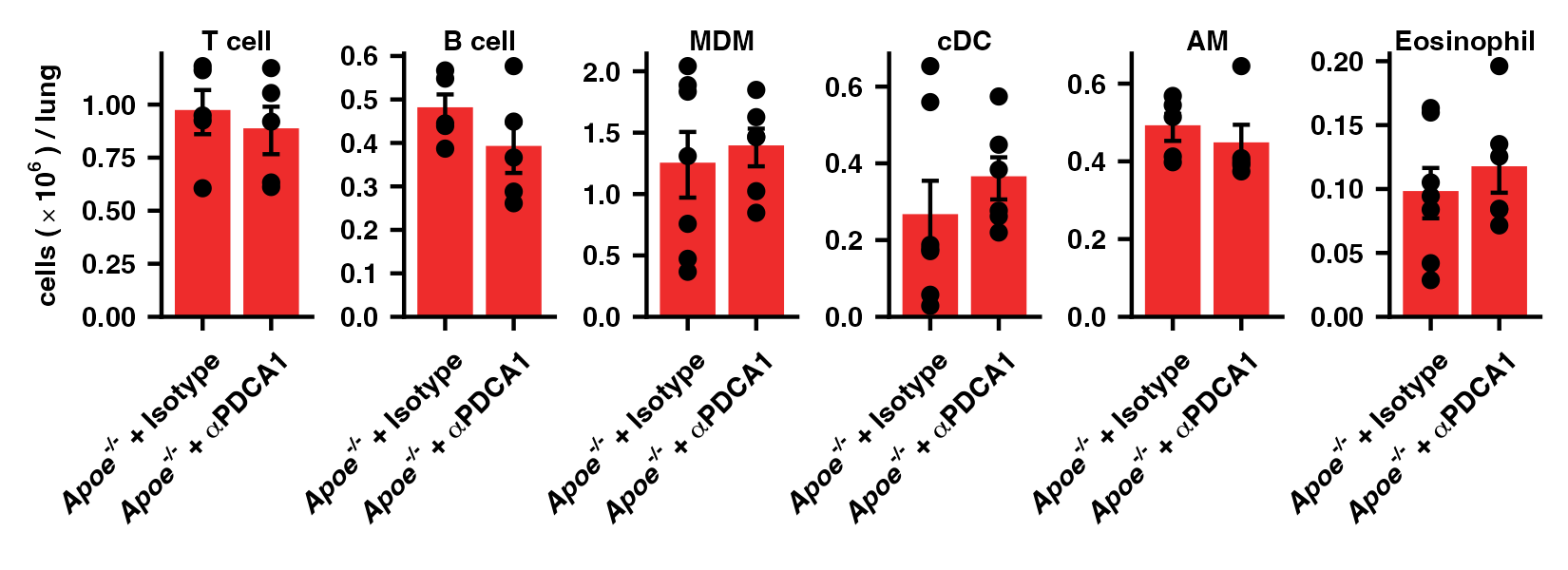

Supplement: S5 Fig — Apoe-/- or B6 mice were placed on a HC diet for two weeks, infected with ~50 CFU H37Rv via aerosol, and maintained on the diet for the entire experiment. Total numbers of the indicated cell types in the lung at day 28 PI following the indicated treatments were measured by flow cytometry. Significance analysis for a difference between treated and control mice was performed using the two-sided Student’s t-test allowing for unequal variances and the Benjamini-Hochberg correction for multiple comparisons. (n = 4–7 mice/group). (TIF) [file ppat.1013267.s005.tif]

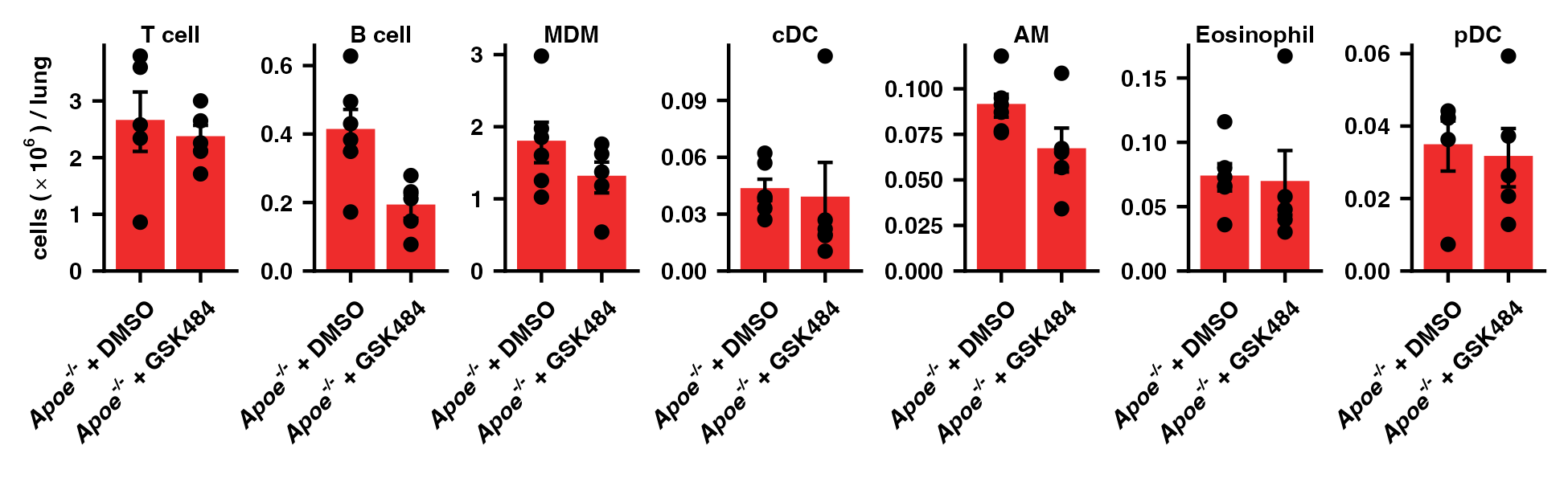

Supplement: S6 Fig — Apoe-/- or B6 mice were placed on a HC diet for two weeks, infected with ~50 CFU H37Rv via aerosol, and maintained on the diet for the entire experiment. Total numbers of the indicated cell types in the lung at day 28 PI following the indicated treatments were measured by flow cytometry. Significance analysis for a difference between treated and control mice was performed using the two-sided Student’s t-test allowing for unequal variances and the Benjamini-Hochberg correction for multiple comparisons. (n = 4–7 mice/group). (TIF) [file ppat.1013267.s006.tif]

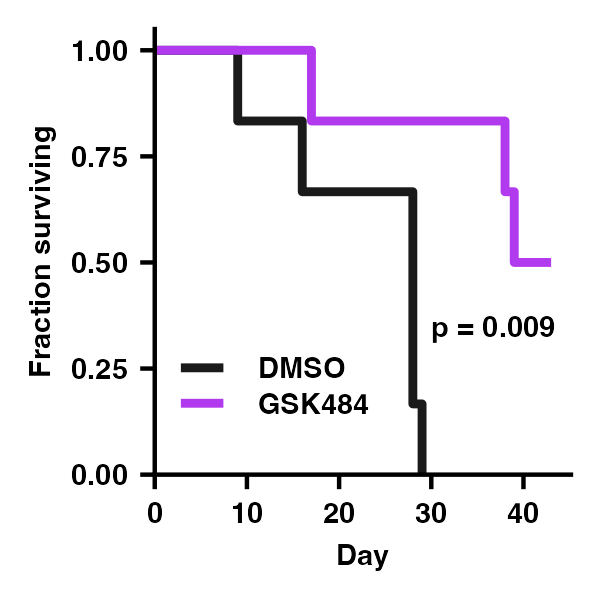

Supplement: S7 Fig — Apoe-/- HC mice were placed on a HC diet for two weeks, infected with ~50 CFU H37Rv via aerosol, maintained on the diet for the entire experiment, and treated with GSK484 or vehicle daily starting at day 7 PI. The fraction of mice surviving to day 40 is plotted. (n = 6 mice/group). Data presented is a replicate experiment for the data presented in Fig 4F. (TIF) [file ppat.1013267.s007.tif]

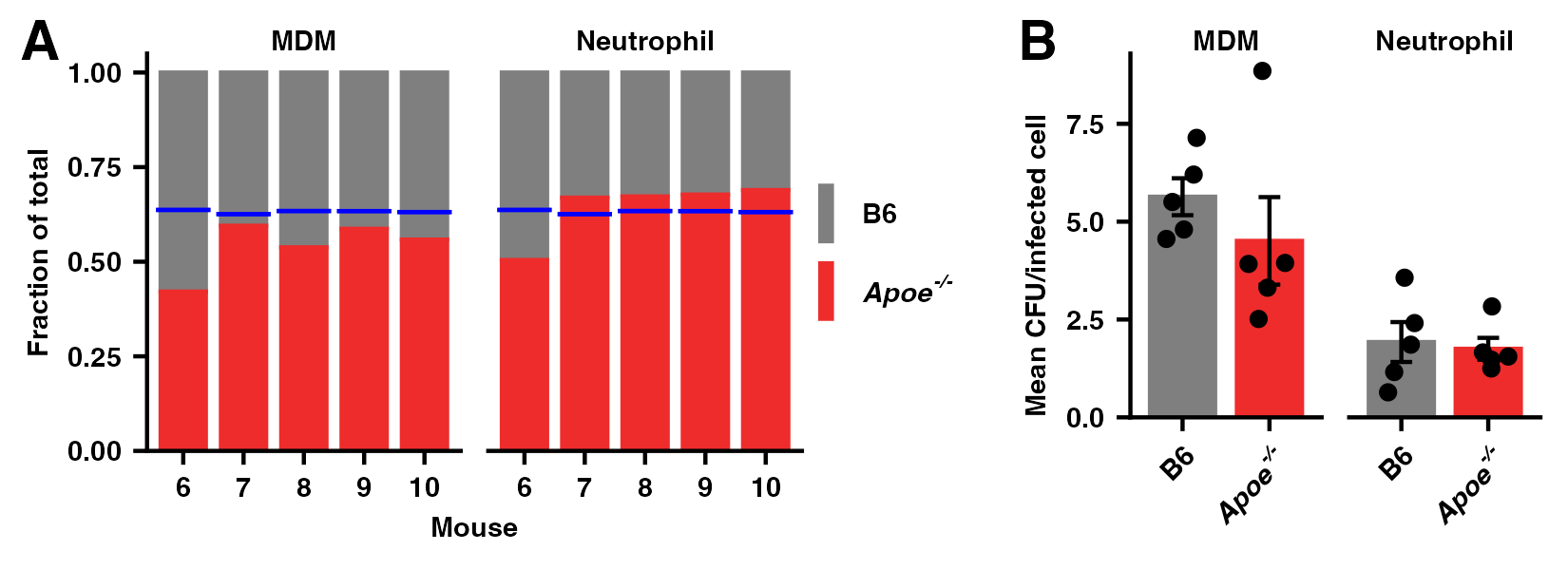

Supplement: S8 Fig — Apoe-/-:B6 mixed bone-marrow chimeric mice on a B6 background were infected via aerosol with ~50 CFU H37Rv. At day 28 PI, monocyte-derived macrophages (MDMs) and neutrophils were isolated by flow sorting. (A) Fraction of MDMs and neutrophils of the indicated genotypes. Blue bars indicate the fraction of circulating CD45 + cells of each genotype prior to infection. (B) Mean bacterial burden in MDMs or neutrophils of each genotype as determined by CFU plating of cells isolated in (A). (TIF) [file ppat.1013267.s008.tif]

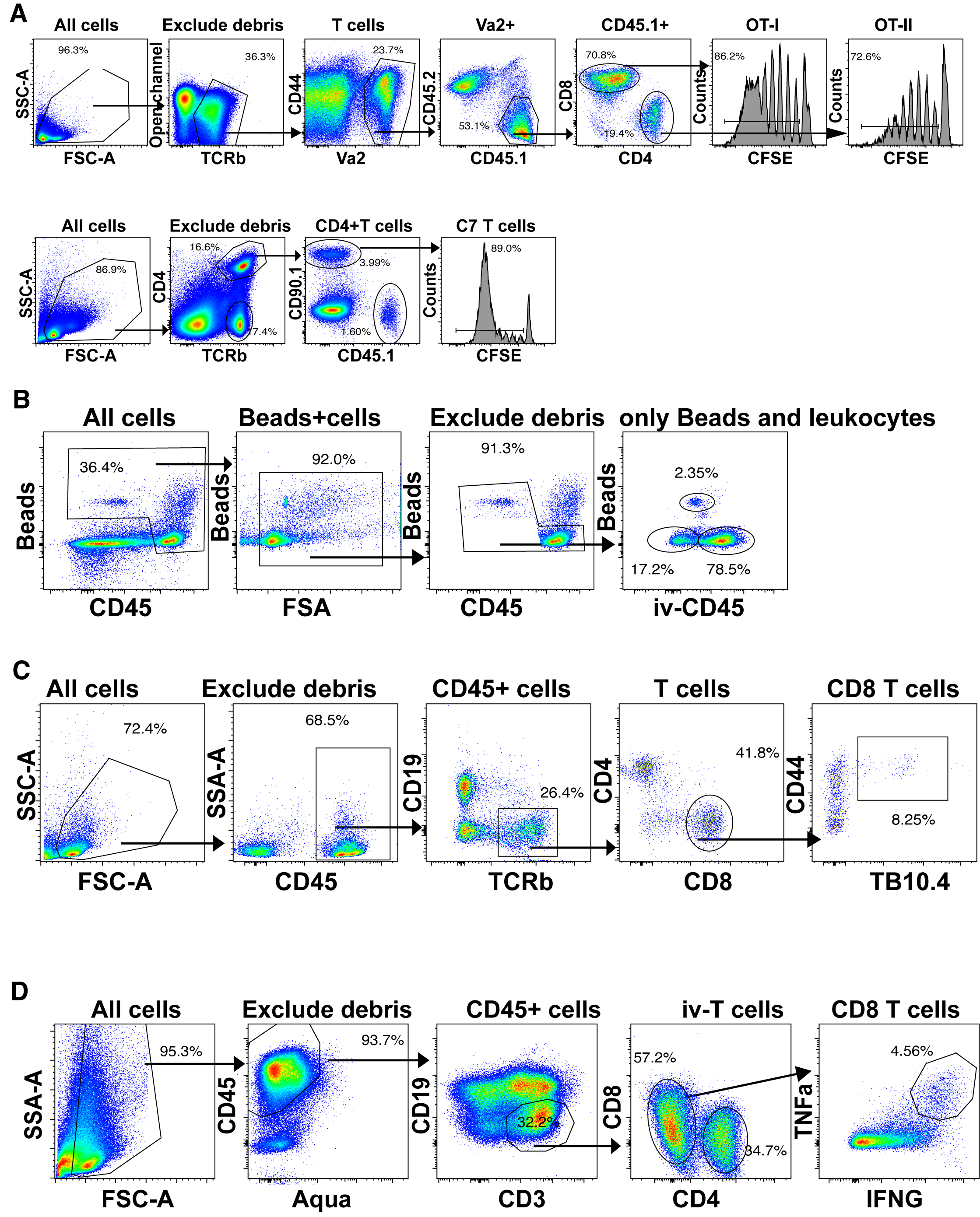

Supplement: S9 Fig — (A) Expansion of adoptively transferred T cells in vivo: The percentage of antigen-specific T cells dividing is computed relative to the total number of single T cells expressing CD45.1 (and CD8 or CD4 in the case of OT-II and OT-I T cells) (B) Absolute counts of total T cells in the lung parenchyma: Single CD45 + cells and counting beads are separated from debris by forward scatter and the absolute number of IV- cells computed relative to the known number of beads added. (C) Fraction of antigen-specific T cells in the lung: The percentage of TB10.4 tetramer+ T cells in the lung was defined relative to the number of single, CD45 + TCRb + CD8 + CD44 + T cells. (D) Ex vivo T cell restimulation: The fraction of CD8 T cells producing both IFNG and TNF was computed relative to the total number of single, live CD3 + CD8 + T cells. (TIF) [file ppat.1013267.s009.tif]

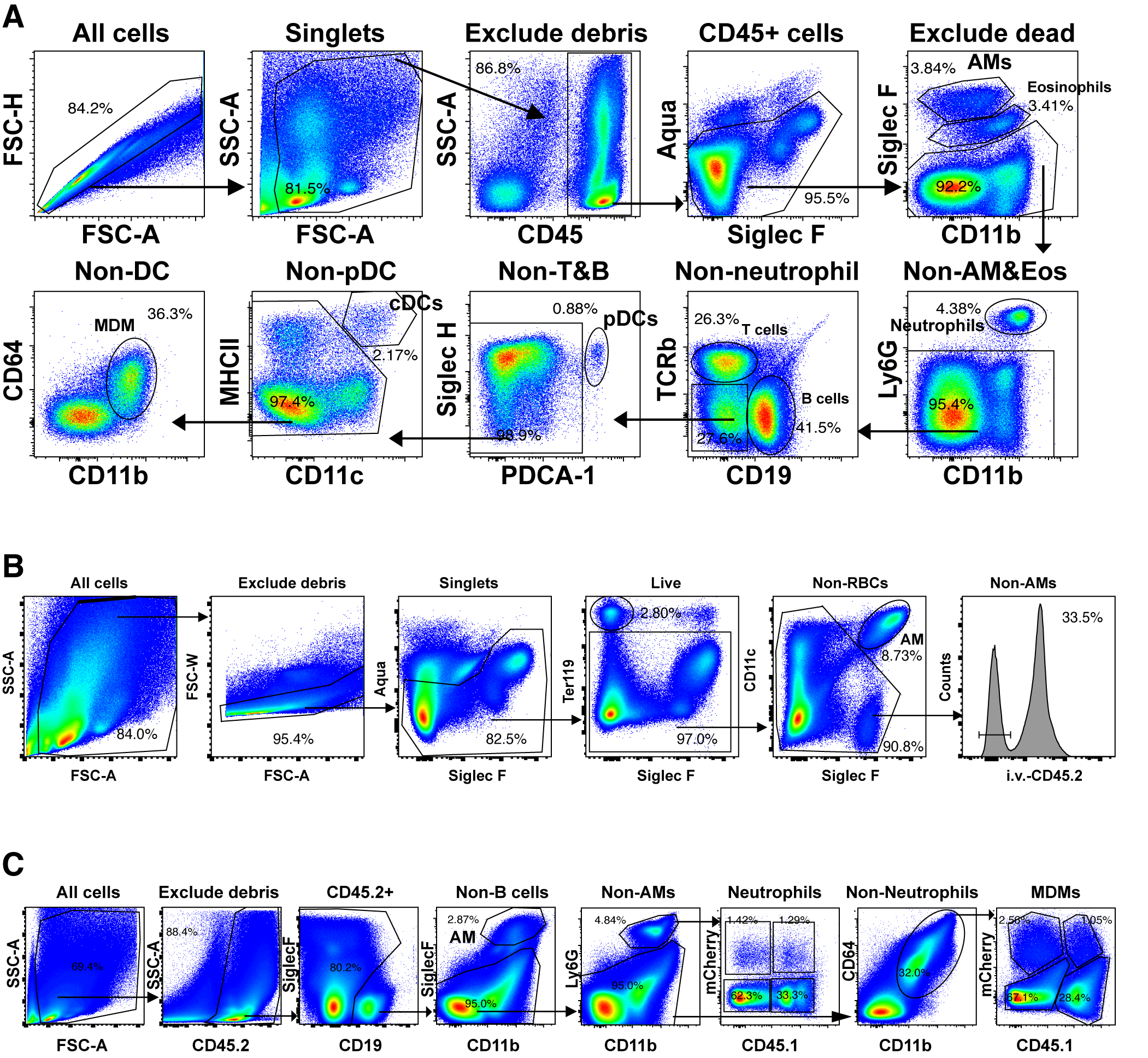

Supplement: S10 Fig — (A) Single, live, CD45 + cells were identified as indicated. (B) Prior to sacrifice, mice were injected with an PE-CD45.2 antibody to mark cells in the circulation. Following digestion of the lung to a single-cell suspension, single, live cells were gated on low Ter119 to exclude red blood cells and low PE-CD45.2 to define the population in the lung parenchyma. In addition, alveolar macrophages (AMs), were defined as SiglecF + CD11c+ cells, regardless of PE-CD45.2 status (IV labeling is unreliable for AMs due to high background autofluorescence.). These two populations were combined and sorted for analysis by single-cell RNA-seq. (C) Mixed bone marrow chimeric mice on a B6 background (B6.SJL-Ptprca Pepcb/BoyJ (CD45.1)) were generated by reconstitution with a 50:50 mixture of B6 (CD45.1.2) and Apoe-/- (CD45.2) bone marrow. To isolate monocyte-derived macrophages (MDMs) and neutrophils single, CD45.2 + , CD19- cells were selected. SiglecF+ alveolar macrophages (AMs) were excluded and infected/uninfected (mCherry + /-) Ly6G+ neutrophils of each genotype isolated by CD45.1 level. Infected/uninfected (mCherry + /-) Ly6G- SiglecF- CD64 + CD11b + MDMs of each genotype were isolated by CD45.1 level. (TIF) [file ppat.1013267.s010.tif]
